# Prevalence of Healthcare-Associated Infections in Patients with Cardiovascular Diseases: A Literature Review

**DOI:** 10.3390/jcm14144941

**Published:** 2025-07-12

**Authors:** Daniela-Mirela Vîrtosu, Angela Munteanu Dragomir, Simina Crișan, Silvia Luca, Oana Pătru, Ruxandra-Maria Băghină, Mihai-Andrei Lazăr, Alina-Ramona Cozlac, Stela Iurciuc, Constantin-Tudor Luca

**Affiliations:** 1 Doctoral School, “Victor Babes” University of Medicine and Pharmacy, 300041 Timisoara, Romania; daniela.cozma@umft.ro (D.-M.V.); silvia.luca0@student.umft.ro (S.L.); oana.patru@umft.ro (O.P.); ruxandra.croicu@umft.ro (R.-M.B.); 2 Institute of Cardiovascular Diseases Timisoara, 13A Gheorghe Adam Street, 300310 Timisoara, Romania; angela.dragomir@umft.ro (A.M.D.); lazar.mihai@umft.ro (M.-A.L.); alina-ramona.cozlac@umft.ro (A.-R.C.); constantin.luca@umft.ro (C.-T.L.); 3 Research Center of the Institute of Cardiovascular Diseases Timisoara, 13A Gheorghe Adam Street, 300310 Timisoara, Romania; 4 Cardiology Department, “Victor Babes” University of Medicine and Pharmacy, 2 Eftimie Murgu Sq., 300041 Timisoara, Romania; iurciuc.stela@umft.ro

**Keywords:** coronary care units, heart failure, infection

## Abstract

This review aims to summarize the global prevalence of healthcare-associated infections in patients with acute heart failure who have been admitted to coronary care units, highlighting the underrepresented burden of infection in this high-risk population. Coronary care units (CCUs) play a pivotal role in the care of patients experiencing acute or decompensated heart failure, offering a highly monitored environment with immediate access to advanced cardiac interventions. The management of heart failure in CCUs involves a multidisciplinary approach that includes hemodynamic monitoring, pharmacologic therapy, respiratory support, and, in selected cases, mechanical circulatory assistance. The early identification of deterioration, rapid therapeutic escalation, and close monitoring of cardiac function are hallmarks of CCU care. However, the complexity and severity of illness in this population are compounded by a high risk of infections, including hospital-acquired pneumonia, bloodstream infections, and device-related infections. These infections not only increase morbidity and prolong hospitalization but also significantly impact mortality and healthcare costs. The immunocompromised state of many heart failure patients—due to poor perfusion, malnutrition, and the use of invasive devices—further elevates their vulnerability. Effective infection prevention, early diagnosis, and targeted antimicrobial therapy are, therefore, critical components of heart failure management within CCUs. This intersection of advanced cardiac care and infection control highlights the need for integrated, multidisciplinary strategies to improve outcomes in this high-risk population.

## 1. Introduction

Coronary care units (CCUs) or coronary intensive care units (CICUs) are hospital wards that specialize in the care of critical cardiac patients who require continuous monitoring and advanced treatment, not just for acute coronary syndrome or post-operative care after coronary artery bypass grafting (CABG) and percutaneous coronary interventions (PCI), but even for decompensated right and/or left heart failure (HF) and malignant arrythmias [1]. Over the past half-century, CCUs have expanded from specialized ischemia arrhythmia monitoring units (CICUs) for acutely ill and medically complex patients with a primary cardiac diagnosis [2]. The CICUs that were transformed from wards focused only on cardiac disease patients admitted to multidisciplinary intensive care units (ICUs). Complications like ventilator-associated pneumonia (VAP), central line-associated bloodstream infections (CLABSI), anesthesia/intubation/sedation complications, stress ulcers, delirium, and the use of inappropriate or false medications, all of which are potentially preventable, are associated with significantly increased in-hospital mortality, morbidity, length of stay, and/or healthcare costs [3]. In Romania, there are five Cardiovascular Institutes with CCUs included in the Cardiology wards (Bucuresti, Cluj, Targu Mures, Iasi, and Timisoara) and every county has an emergency hospital CICUs, considered to be level-II ICUs, where cardiology specialists and physicians specializing in anesthesiology and intensive care are working together, as a team.

HF can be described as a syndrome characterized by reduced functional capacity, repeated hospitalizations, poor quality of life, and high mortality [4]. The estimated prevalence of HF is about 64 million patients globally, thus being considered a real pandemic [5], with a high variability between countries and a median prevalence of HF per 1000 people estimated at 17, ranging from 10–14 per 1000 in Spain and the Netherlands to 20–39 per 1000 people in Slovenia, Lithuania, and Germany [6]. In Central European countries like Hungary, Montenegro, Slovakia, and Slovenia, the prevalence of heart failure was relatively high in 2017, at around 12 per 1000 people, while the lowest age-standardized prevalence rate in Europe was observed in an Eastern European country, Latvia (5 per 1000 people) [7]. In Romania, 4.7% of patients over 35 years of age were diagnosed with HF [8]. Acute heart failure (AHF) is defined as new (de novo) or worsening symptoms and signs of HF, which are mostly related to systemic congestion, this being the most frequent cause of unplanned hospital admission in patients over 65 years of age. In the presence of underlying structural or functional cardiac dysfunction, one or more precipitating factors can induce AHF, although sometimes de novo HF can result directly from the onset of a new cardiac dysfunction issue, most frequently in acute coronary syndromes [9]. As with de novo AHF, decompensation of chronic HF can occur in patients with atrial fibrillation/flutter, valvular heart disease, and dilated cardiomyopathy [10]. Symptoms like fatigue, breathlessness, or swelling of the ankles, and signs like peripheral edema, elevated jugular venous pressure, or pulmonary crackles due to reduced cardiac output and/or high intracardiac pressure are typical for HF [11]. Arterial hypertension is one of the major causes of cardiovascular morbidity and mortality in Europe, with HF, left ventricular hypertrophy, and coronary artery disease being among the most common complications [12]. More than 60% of patients with congestive HF are initially diagnosed in the emergency department [13]. More than half of HF patients will be readmitted to the hospital within 6 months of discharge, leading not only to increased health care expenditures but also to functional decline, iatrogenic injuries, and hospital-acquired infections (HAIs) [14]. According to HF guidelines, cardiac resynchronization therapy (CRT) using triple-chamber cardiac devices is the current standard treatment in HF patients, and all patients undergoing CRT therapy will be monitored post-procedurally in ICUs [15].

Given the limited availability of data focused exclusively on CCUs, this review aims to consolidate the global evidence on HAI prevalence in patients with AHF, including both general ICU data and CCU-specific studies where available. While this is the aim of this review, the availability of direct data in this specific setting is limited. Therefore, the initial sections of this manuscript examine HAIs in broader cardiovascular and ICU populations. This context enables a more comprehensive understanding of risk profiles, infection types, and surveillance gaps, ultimately supporting a more informed discussion of the challenges specific to CCUs.

## 2. Healthcare-Associated Infections

HAIs, also known as nosocomial infections or hospital-acquired infections, are one of the major threats to hospitalized patients. HAIs represent one of the most frequent complications of hospitalization worldwide, with an annual incidence ranging from approximately 5 to 15% of all hospitalized patients [16]. An HAI, according to Romanian Ministry of Health Order no. 1101/2016, can be defined as an infection contracted in sanitary units with beds (public or private) and refers to any infectious disease that can be clinically and/or microbiologically recognized with an epidemiological proof of contacting it during hospitalization/medical acts or medical procedures, which can affect either the patient, due to the medical care received, or the medical staff, due to the patient’s activity. The incubation period of an HAI is related to the period spent under medical care in the respective unit, and symptoms of the disease may appear during the hospitalization period (after 48 h) or immediately after the time of discharge (except for surgical site infections involving invasive devices, when an HAI can be considered to appear up to one year after surgery [17].

The coordination of the European surveillance of HAIs was transferred to the European Centre for Disease Prevention and Control (ECDC) in 2008. Promoting the prevention of HAIs and antimicrobial resistance in European ICUs, monitoring the burden of HAIs and antimicrobial resistance in European ICUs, and describing the epidemiology of HAIs in European ICUs are some of the specific objectives at the European level [18].

The HAIs identified in healthcare facilities can be classified into 3 major categories:

1. Device-related infections (CLABSI, VAP, UTI, and infection of a prosthetic device);
2. Non-device-related infections (HAP—hospital-acquired pneumonia, MDRO—MRSA—multidrug-resistant organisms—methicillin-resistant Staphylococcus aureus, Clostridium difficile infections (CDI), VRE—vancomycin-resistant Enterococci, and Gram-negative or ESBL—extended-spectrum beta-lactamase-producing);
3. Procedure-related infections (transplant-associated infections, BSI—bloodstream infection, SSI—surgical site infection, and septicemia).

Pathogens responsible for HAIs include bacteria, viruses, and fungi [17]. The most common route of transmission for the pathogens associated with HAIs is through direct or indirect contact (multidrug-resistant bacteria such as MRSA, ESBL, VRE, C. difficile, and rotavirus). Droplet transmission may occur when microorganisms are transmitted from the respiratory tract by large droplets (like Influenza). Airborne transmission involves the transmission of organisms from the respiratory tract by small droplets that travel long distances (like the SARS-CoV-2 virus) [19]. To prevent, monitor, and control HAIs, the 2012/506/EU Decision lays down the case definitions for doctors in order to diagnose and report communicable diseases to the European Community network. In addition to the clinical manifestations and physical examination, routine blood tests, including complete blood counts, metabolic panels, inflammatory biomarkers, and blood gases, along with microbiological tests, can be useful to evaluate HAIs.

Recent data from the World Health Organization indicate an annual occurrence of 8.9 million HAIs in acute and long-term care facilities. The majority of these cases are reported in ICUs [20]. The prevalence of HAIs in high-income countries is 7.5%, although others have reported rates of 5.7–7.1% in Europe and 4.5% in the US, while in low- and middle-income countries, the prevalence rate ranges between 5.7% and 19.2% [21]. The prevalence rate differs from one country to another, depending on the possibilities of infection prevention and control [22]. However, the exact burden of HAIs in each country is not yet known [23]. Moreover, reported HAI rates may be influenced by hospital-level goals or quality improvement targets. For example, some institutions may implement strict policies to limit the use of urinary catheters in order to reduce the incidence of CAUTIs, thereby reporting artificially low infection rates. Similarly, selective surveillance or under-reporting—especially in regions with limited infrastructure—can further obscure the true prevalence and also complicate interfacility or international comparisons.

A report of the results of an International Nosocomial Infection Control Consortium (INICC) surveillance study carried out between 2012 and 2017, which included 523 intensive care units from 45 countries in Latin America, Europe, the eastern Mediterranean, Southeast Asia, and the western Pacific shows a higher rate of HAIs compared to the rates reported by ICUs from the Centers for Disease Control and Prevention’s National Healthcare Safety Network (CDC-NHSN). In medical–surgical ICUs, the pooled CLABSI rate was higher (5.05 vs. 0.8 per 1000 central line-days), the VAP rate was also higher (14.1 vs. 0.9 per 1000 ventilator-days), and the rate of CAUTI was also higher (5.1 vs. 1.7 per 1000 catheter-days) [24].

The infection prevention and control team (IPC team) has an important role in Romania in order to coordinate the prevention, surveillance, and control of hospital-acquired infections, according to Order no. 1101/2016 regarding the approval of the norms of supervision, prevention, and limitation of infections associated with medical assistance in healthcare units, along with every single employee of the healthcare facility, functioning as a whole, not independently [17]. Each medical facility must develop its own annual infection prevention program with the help of the IPC team. In addition, each employee has responsibilities laid down in their job description regarding the prevention and control of HAIs [25]. Hand hygiene is the most important aspect of infection control and the prevention of healthcare-associated infection. In addition to hand hygiene, effective strategies to reduce HAIs include the implementation of antimicrobial stewardship programs to limit inappropriate antibiotic use and reduce resistance; strict protocols for the insertion, maintenance, and timely removal of invasive devices such as urinary catheters and central venous lines; the use of contact precautions and cohorting for patients who are colonized or infected with MDROs; staff education and regular training on infection control procedures; routine environmental cleaning and disinfection protocols; and the use of checklists for high-risk procedures. Surveillance systems and feedback loops can also help identify outbreak patterns early and guide corrective actions [19]. Infection prevention and control programs act as quality improvement activities to develop, implement, and monitor protocols and interventions that aim to limit HAIs [26].

## 3. Prevalence of HAIs in Patients with Cardiovascular Diseases

A systematic review and meta-analysis performed between 2000 and 2001 that included more than 29 million participants showed a universal HAI rate of 0.14%, increasing by 0.06% annually. The highest rate of HAIs was in Africa (0.27%), while the lowest prevalences were in America and the western Pacific area (0.09%). Europe had a point prevalence of 0.11%, and in Asia, the prevalence was 0.12%. Furthermore, a higher prevalence rate was found in central Africa than in other parts of the world. According to WHO data, the HAI rate is 25% in developing countries and 5–15% in developed countries [27].

### 3.1. Prevalence of HAI in Patients with Cardiovascular Diseases from the African Continent

The only study found from the African continent is a cross-sectional study on patients admitted to general ICUs in Uganda. The study included 111 adult patients admitted to two of the ICUs in Uganda and concluded that the HAI prevalence was 28.82% [28].

### 3.2. Prevalence of HAI in Patients with Cardiovascular Diseases from the American Continent

Very little is known about the incidence and outcomes of HAIs among patients hospitalized with common cardiovascular conditions in America. In a study covering the period between 2008 and 2015, a team from the USA analyzed the National (Nationwide) Inpatient Sample (NIS)’s database and identified 159,021 hospitalizations with at least one HAI from the total number of 17,889,852 hospitalizations (with an overall HAI rate of 0.88%). Of these, 49.6% of patients were diagnosed with HF, 20.4% with acute myocardial infarction (AMI), 10.5% with CABG, 18.6% with cardiogenic shock, and 11.9% with atrial fibrillation or flutter. The most common infections were Clostridium difficile infections (75.4%), followed by catheter-associated UTIs (15.1%), VAP (7.9%), and CLABSI (3.1%). Of all pathologies, cardiogenic shock was most commonly associated with HAIs, as recorded in 4% of cases [29].

In US acute care hospitals, the HAI rate published by Marchetti in 2013 affected 4.5% of admissions [30]. A slightly higher rate of 4.87% was found in the report by Klevens et al. from 2002 [31].

Between January 1986 and December 1995, in a 55-bed cardiothoracic ICU in the US, a total of 40,207 patients were admitted to the CICU during the 10-year study period. In the study, 681 patients with 804 episodes of nosocomial BSIs were identified, yielding an HAI prevalence rate of 1.69% [32].

The results of a multicenter observational study of the Cardiothoracic Surgery Clinical Trials Network from 2010, which was carried out with cardiac surgery patients from the US, confirmed that 2.8% of patients experienced a major HAI during the index hospitalization. The most common HAIs were pneumonia (48%), sepsis (20%), and C. difficile colitis (18%) [33]. A retrospective review based on the collected data of patients who underwent cardiac surgery at a single institution in Baltimore (USA) between 2012 and 2018 found that 4% of all patients developed a postoperative HAI [34]. In another retrospective study performed in Boston (USA), hospital-acquired infections following cardiac surgery had a similar incidence rate of HAI of 4.2% [35]. In contrast, in a recent study performed in St. Louis, USA, in a university-affiliated teaching hospital, 21.7% of patients acquired at least one nosocomial infection following cardiac surgery [36]. Furthermore, in a study conducted in Brazil between 2012 and 2018, the prevalence of HAIs in patients who underwent cardiac surgery was 22.6% [37].

The NIS from New Jersey was queried for the period between 2003 and 2007. The conclusion was that the total infection rates significantly increased after elective surgery delays like CABG, and 5.73% of patients had been diagnosed with HAIs [38]. Furthermore, from 2011 to 2013, in a study of 998 hospitals in America, 2.83% of patients experienced pneumonia after CABG [39].

Data on STEMI cardiogenic shock patients (STEMI-CS) sourced using the NIS database in the US from the years 2005–2014 showed that 19.1% of patients admitted with STEMI-CS developed HAIs, with a descending trend for HAIs. The most common HAIs were UTIs (9.2%), followed by VAP (6.8%), CLABSI (1.5%), bacteremia (1.5%), skin-related infections (1.5%), and CDI (1.3%) [40]. In an older study from Brazil on AMI patients, performed between 1996 and 1999, 5% of patients developed infectious complications [41]. Furthermore, an observational prospective case series study performed from 2006 to 2011 in a public hospital in Rio de Janeiro, Brazil, showed that 35.09% of infective endocarditis (IE) cases were HAIs [42].

### 3.3. Prevalence of HAIs in Patients with Cardiovascular Diseases from the Asian Continent

Surveillance for HAIs, conducted among all patients admitted to adult ICUs from 2003 through 2005 in China, showed an incidence rate of 12.1% in the medical ICU, 14.7% in the surgical ICU, and 16.7% in the mixed medical and surgical ICU (*p*-value > 0.05) [43].

In a study of a hospital in China from 2018, of those patients undergoing open heart surgery who had been transferred to a cardiac surgical ICU, 6.54% of them acquired a microbiologically documented HAI: 2.24% were SSIs, while 0.75% were UTIs [44]. The records of patients from India who underwent cardiovascular surgery during 2013 and 2014 showed that 4.6% developed HAIs after cardiac surgery [45]. In a cross-sectional descriptive-analytic study (2013–2017) of patients who underwent surgery at the Department of Cardiac Surgery in Iran, the incidence of nosocomial infections following cardiac surgery ranged from 17% to 23% [46]. Moreover, 20.97% of patients undergoing valve replacement surgery in China from 2018 to 2019 subsequently experienced nosocomial infections [47]. Another study from China, published in 2018, studied a cohort of cardiovascular surgery patients and concluded that 7.8% of patients developed HAIs; the HAI rates following surgeries for valve replacement and CABG were 5.5% and 13.6%, respectively. The HAI rate following the surgical repair of an aortic aneurysm or aortic dissection was 16.8%. The rates of the different main types of HAI were 0.7% for an SSI, 0.8% for a CVCRI, and 1.1% for a UTI [48].

Hospital data from patients with atrial fibrillation (AF) and without AF were collected from the Department of Cardiology, First Affiliated Hospital of Shantou University Medical College, Shantou, China, from 2009 to 2011. The results showed that 25.64% of patients with AF had HAP, compared to patients without AF, who maintained a rate of only 3.66% [49].

A retrospective study, conducted in 2020, on patients from Japan who were diagnosed with IE found a 33.5% rate of HAIs [50]. In a meta-analysis that included 23 articles from China, the United States, South Korea, France, Australia, and Croatia concerning patients with extracorporeal membrane oxygenation (ECMO), an overall prevalence rate of 31.57% for HAIs was found [51]. Moreover, a retrospective cohort study conducted in a single tertiary referral center in Korea between January 2010 and December 2018, which included adult patients who required extracorporeal cardiopulmonary resuscitation for in-hospital cardiac arrest, concluded that 23.3% of patients developed HAIs from patients with ECMO. Pneumonia was the most common type of HAI, followed by catheter-related infection [52].

### 3.4. Prevalence of HAIs in Patients with Cardiovascular Diseases from the Australian Continent

In a prospective cohort study conducted from 2000 to 2006 in 61 Australian centers, in 28 countries, cardiac device infective endocarditis (CDIE) was diagnosed in 6.4% of the total cohort of patients with defined infective endocarditis [53]. In studies on patients post-cardiac surgery who were included in a cardiac surgery registry (2001–2011), in the 16 hospitals in Australia, pneumonia rates varied from 0.7% to 12.4% across hospitals [54]. In a study published by Harmon et al. in 2020, 23% of patients from 36 centers in Europe and Australia who were admitted to ICUs after out-of-hospital cardiac arrest developed pneumonia, and 4% of them had bacteremia with a clinically relevant pathogen [55].

### 3.5. Prevalence of HAIs in Patients with Cardiovascular Diseases from the European Continent

In Europe, every year, more than 4 million people develop HAIs, resulting in 16 million (6%) additional hospital days and approximately 37,000 deaths [16]. The overall prevalence of HAIs in acute care hospitals in Europe was 6.0%, as reported in a point prevalence study from 2011 to 2012, and the most frequent HAI types were surgical site infections and pneumonia. By far the highest prevalence of HAIs in 2011–2012 was found to be in intensive care, with a rate of between 15 and 20%. The most prevalent microorganism involved in the etiology of HAIs in Europe in 2011 and 2012 was Escherichia coli in 15.9% of cases, followed by Staphylococcus aureus and Enterococcus [56].

The European Centre for Disease Prevention and Control (ECDC) surveillance report from 2021 concluded that 15.6% of patients who stayed in an intensive care unit for more than two days presented at least one of the ICU HAIs under surveillance, such as pneumonia, bloodstream infections, or urinary tract infections [57].

An ECDC point prevalence report from 2022–2023 in acute care hospitals showed that the overall prevalence of HAIs was 7.0% and the most frequent HAI types were surgical site infections and pneumonia. The prevalence of HAIs in the period from 2022 to 2023 that were reported in intensive care exceeded 20% [58].

The data obtained over a period of 10 years from 2005 to 2015, on patients from Spain who were admitted to an ICU with acute cardiac disease, showed that 5.1% of the patients had one or more infections [59]. Infection complications during their ICU stay occurred in 17% of AHF patients from Switzerland. A French, multi-center study performed on ICU patients with AHF revealed that the infection rate was 20%. Moreover, a Finnish multi-center study that investigated hospitalized patients with AHF reported infections in 24% of cases [60]. In the EuroHeart Failure Survey II on AHF patients, infections were diagnosed in 18% of hospital admissions [61].

In total, 36.5% of all cases of ECMO patients hospitalized between 2012 and 2016 in a single cardiac center from Turkey developed a nosocomial infection, with a rate of 49/1000 ECMO days. Infection was associated with more than 5 days of ECMO duration. The most frequent infection site was the lower respiratory tract (14.3%), while the most common isolated organisms were Klebsiella (8.7%) and Streptococcus (4.8%) species [62].

In 2005–2009, all hospitals in Norway that performed CABG procedures evaluated all patients undergoing CABG surgery over 3-month periods for SSIs, and identified 5.1% sternal infections and 8.9% nosocomial infections at the harvest site. Over 95% of infections occurred after hospital discharge [63]. A prospective cohort study performed in a hospital in Athens, Greece, which included adult patients who underwent coronary artery bypass grafting with no valve surgery and without the use of cardiopulmonary bypass over a period of 3 years, concluded that 2.7% of the studied patients acquired microbiologically documented nosocomial infections after off-pump CABG procedures [64]. Elsewhere, 38.5% of patients developed any type of infection after cardiac surgery, as reported in a study performed on patients with structural heart disease and/or stable coronary vessel disease, who were scheduled for elective cardiac valve and/or CABG surgery at the Department of Cardiac Surgery of the Medical University of Vienna (Austria) [62]. The frequency, risk factors, and characteristics of infections were evaluated in patients after off-pump CABG surgery in a prospective study performed during 2004–2005 in Athens, Greece, where 5% of patients developed postoperative infections [65].

In Spain, HAIs occurred in 9.2% of patients who underwent cardiovascular surgery between 1993 and 1994 [66], in 15.5% of participants consecutively undergoing a MHS (major heart surgery) procedure [67], and in 10.1% of adult patients undergoing cardiac surgery at the Hospital Clinico Universitario de Valladolid, Spain, between 2011 and 2016 [68]. In Greece, infections occurred in 13.95% of patients who underwent open heart surgery between 2006 and 2008 and who were enrolled in a prospective study in the cardiovascular intensive care unit (CVICU) of the University Hospital of Ioannina [69], while 5.0% of patients in another study in Greece developed microbiologically documented nosocomial infection after open cardiac surgery [70]. In a prospective, randomized, double-blind, placebo-controlled clinical trial conducted in Amsterdam, the Netherlands, between 2003 and 2005, the incidence of nosocomial infection in the chlorhexidine gluconate group and placebo group was 19.8% and 26.2%, respectively, in patients undergoing elective cardiothoracic surgery [71]. In a study population that included patients from a tertiary care facility in Italy who were undergoing cardiac surgery between 2005 and 2006, 9% of patients developed an HAI [72]. In a study group that included all consecutive patients who underwent cardiac surgery over a 10-year period from 1997 to 2007 in France, in 1.7% of the cases, SSIs were identified [73]. Moreover, at least one nosocomial infection was detected in 4.4% of patients with MHS in 25 hospitals from Europe [74]. Another study that assessed the prevalence of infections in patients undergoing MHS in 13 European countries concluded that the overall prevalence of infection (VAP) was 26.8% [75]. During the study period, 9.9% of patients undergoing MHS in ICUS in seventeen hospitals from seven European countries developed one or more nosocomial infections [76] (Table 1).

For a more comprehensive perspective, national data on the prevalence of HAIs in patients with cardiovascular diseases in Romania are presented in conjunction with findings from the other countries presented above. Subsequent to the enactment of Order no. 1101 from 2016 [77] regarding the approval of the norms of surveillance, prevention, and limitation of HAIs in the health units in Romania, the reporting rate of HAIs has constantly increased, ranging from 0.2 to 0.25% in an official Romanian report [17], up to 2.6% in a European report from 2018 [78], but the prevalence rate is still far from the European average of 7.1% [17], with HAIs representing a much-underestimated pathology.

In a Romanian ICU study conducted on a western Romanian university hospital and performed between 2012 and 2013, 35.9% of patients with non-infectious pathology developed HAIs during their hospital stay, with a predominance of HAI pneumonia (38.42%/22.05%) and BSI (32.38%/25.19%) (Table 2) [79].

In 2020, Tereanu et al. reported an incidence of HAIs in Romania of 0.44% in 2016, while the EU average was almost ten times higher, with a value of 5.2% [80].

According to the National Center for Statistics and Informatics in Public Health, of the total number of HAIs reported out of all patients in 2017, the most frequent were digestive (41%), followed by respiratory (18%) and urinary (13%) infections and infected surgical wounds (12%). Clostridium difficile infection showed an ascending trend since 2011, with a similar evolution of the overall HAI rate, rising from 0.33% in 2015 to 0.63% in 2019 [81].

In a cross-sectional study utilizing data provided by the Mures Public Health Directorate from all 7 public hospitals that examined HAIs from 2017 to 2021, the hospitals reported an overall HAI rate of 0.44%. The most frequent HAIs were reported by ICUs in 48.4% of cases. The medical departments reported the highest prevalence of HAIs, with a value of 48.25%. In the same study, the most common infections included bronchopneumonia (25.3%), enterocolitis with C. difficile (23.3%), sepsis, surgical wound infections, and UTIs [82]. The National Center for Surveillance and Control of Communicable Diseases in Romania published in 2020 that the HAI rate in Romania was 1.04%, with an ascending trend from 2012 to 2020 (2.8 times higher in 2020 compared to the minimum rate level reported in 2012), accelerating after 2016. Respiratory infections were the most frequent related infections, found in 37% of cases, doubling their number in a report recorded in 2019 [83].

A report from 2023 by the Ministry of Health in Romania and the State Sanitary Inspection Department, in which 333 medical facilities from Romania were analyzed, showed that 1.2% of patients undergoing medical procedures developed a healthcare-associated infection during hospitalization. The highest rate of HAIs was found in intensive care and surgery, mostly due to venous catheterization, major surgical procedures, or exhaustive antibiotic consumption [84].

## 4. HAIs Prevalence in Coronary Care Units

Limited data are available on HAIs in patients in CCUs, as reported in studies published in the medical literature (Table 3). Contemporary CICUs show an increasing prevalence of non-cardiovascular comorbidities and multisystem organ dysfunction. In patients with mechanical circulatory support, the rates of infection vary along with duration of use and the type of mechanical circulatory support device, with reported incidences ranging from 1% for intra-aortic balloon pumps to nearly one-third of patients requiring extracorporeal membrane oxygenation support [85].

Infection rates in various ICUs in a hospital in the USA between 1981 and 1983 ranged from 1.0% (cardiac surgery ICU) to 23.5% (medical/surgical ICU). Rates of ICU-acquired infection ranged from 0.8% (cardiac surgery ICU) to 11.2% (medical/surgical ICU) [86].

In a study published in 1986, only 1.9% of patients in the CCU were infected out of the total number of patients admitted between May and July 1984 to the Detroit Receiving Hospital (USA) [87].

To describe the epidemiology of nosocomial infections in CCUs, Richards et al. analyzed data from CCU patients in the USA (1992–1997). Urinary tract infections (35%), pneumonia (24%), and primary bloodstream infections (17%) were almost always associated with the use of an invasive device (93% with a urinary catheter, 82% with a ventilator, and 82% with a central line, respectively). The mean overall patient infection rate was 2.7 infections per 100 patients [88]. In a retrospective, case-control clinical trial on patients with AMI who were admitted to the CCU in a hospital in Brazil between 1996 and 1999, 5% of patients were diagnosed with infectious complications. The most frequent infections were pulmonary (63%), followed by UTIs (37%) and positive blood cultures with no identifiable site of infection (8%) [89].

In a retrospective cohort study from 2002–2003, performed in the CCU of a single tertiary medical center in the USA and including patients who required invasive mechanical ventilation for more than 48 h, the authors concluded that 18.5% of patients developed VAP. The incidence of VAP was 36.3 (95% confidence interval, 21.1–58.1) per 1000 days of mechanical ventilation [88].

Moreover, 4.11% of patients admitted to a cardiac intensive care unit in India developed HAIs, as reported in a study published in 2008 [90].

A point-prevalence survey from USA hospitals, covering the period from 2011 to 2015, showed a 6.27% (115/1834 patients) prevalence of HAI in critical care units [91].

In the coronary ICU of a cardiac hospital in Qatar, the CLABSI rate was 2.82/1000 central line days in 2015 and 3.11/1000 central line days in 2016 (a higher rate than the National Healthcare Safety Network’s 50th percentile benchmark of 0.8) [92].

Another study evaluated adults receiving veno-arterial extracorporeal membrane oxygenation (VA-ECMO) for more than 48 h for cardiac arrest or severe cardiogenic shock from January 2013 to December 2018 within the CICU of a 1200-bed tertiary hospital in Madrid, Spain, concluding that 42% of patients treated with VA-ECMO for up to 48 h developed at least one infection, with an infection rate of 0.92/1000 VA-ECMO days [93].

In a retrospective study from Iran, the researchers compared the incidence of nosocomial infections in Sina Hospital before and after the COVID-19 period (2019 and 2020). Before COVID-19, the incidence of nosocomial infections in the CCU was 0.98%, while during the COVID-19 period, the incidence rate was higher (3.44%) [94].

In a study in Turkey on 500 patients hospitalized in the coronary intensive care unit for more than 48 h between January 2019 and December 2020, the most common detected infection type was CLABSI, which was found in 79.1% of cases, followed by CAUTI in 18.7% of cases, and VAP in 6.25% of cases. Gram-negative Bacillus infections accounted for 70.8% of the causative agents, Klebsiella being the most frequently isolated bacteria [3]. Out of the total samples (2478) obtained during January 2020 and December 2021 from patients admitted to CCUs from cardiology wards after surgery in India, 271 (10.93%) patients developed HAIs. Acinetobacter was the most frequent pathogen to be isolated in patients with lower respiratory tract infections and bloodstream infections, while *E. coli* was harvested from UTIs [95].

The medical data of nosocomial patients in China were collected during the second and third quarters of 2023. During the second quarter, the incidence of CAUTI was 0 per 1000 urinary catheter days in CCU. During the third quarter, the incidence of CAUTI was 2.98 per 1000 urinary catheter days in CCU [96].

In a systematic review published in 2023, only 3 studies from a total of 400 records included in the 2000–2021 analysis concerned CCUs, with a point estimate for global prevalence of 0.10%, compared to 140 studies concerning ICUs, with a point estimate of 0.68%, concluding that central African hospitals had higher rates of HAIs than in other parts of the world [27].

**Table 3:** Prevalence of HAIs in CCUs.

Continent	Country	Population	No of Tested Persons	Seroprevalence of HAIs	Reference
AMERICA	USA	CCU patients	-	1.9%	Chandrasekar PH, 1986 [87]
AMERICA	USA	CCU patients	227,451	2.7%	Richards MJ, 1998 [88]
AMERICA	USA	CCU patients	1834	6.27%	Magill S, 2018 [91]
AMERICA	Brazil	AMI patients	1227	4.88%	Richards MJ, 1998 [88]
ASIA	Iran	CCU patients	10,553	0.98–3.44%	Mornese Pinna S, 2023 [93]
ASIA	India	CICU patients	3161	4.11%	Pawar M, 2008 [90]
ASIA	India	CCU patients	2478	10.93%	Yadav S, 2024 [95]
EUROPE	Spain	CICU VA-ECMO patients	69	42.0%	Mornese Pinna S, 2023 [93]

CCU = Coronary Care Unit; AMI = acute myocardial infarction; CICU = Coronary Intensive Care Unit; VA-ECMO = venoarterial extracorporeal membrane oxygenation.

There is no published study on the prevalence of HAIs in CCUs in Romania. ECDC PPS reports from 2011–2012 and 2022–2023 included a small number (10 in 2011–2012, 53 in 2022–2023) of hospitals in Romania. The reports showed that the overall prevalence of HAIs in Romania in 2011–2012 was around 2.6%, similar to the prevalence found in 2022–2023. The HAI prevalence in medical ICUs in 2011–2012 (1.2%) was similar to that in 2022–2023 (1.1%). By far the highest prevalence of HAIs in 2011–2012 was found in intensive care (between 15 and 20%), and the most prevalent microorganism implicated in the etiology of HAIs in Romania in 2011–2012 was Staphylococcus aureus (18.9%), followed by Klebsiella (13.5%). The prevalence of HAIs in 2022–2023 in intensive care exceeded 20%. The most prevalent microorganism implicated in the etiology of HAIs in Romania in 2022–2023 was Clostridium difficile, which had by far the highest prevalence (26.0%), followed by Klebsiella (13.5%) [56,58].

## 5. Discussion 

This review highlights the significant variability in HAI prevalence among cardiovascular patients across different regions, with a notable lack of data focused specifically on AHF patients admitted to CCUs. The vast majority of published studies provide prevalence data from general ICUs or surgical wards, with little focus being on CCUs or even medical ICUs. In particular, no studies were identified regarding HAI prevalence in CCUs from Romania.

In the United States, CCUs are typically a subset of ICUs that are dedicated to the care of critically ill cardiac patients and are often located in hospitals that perform cardiothoracic surgery. In contrast, in Romania, CCUs are integrated within cardiology departments, caring for patients with acute coronary syndromes, post-operative cardiac surgery or interventions, AHF, and arrhythmias. These differing institutional frameworks likely influence not only the types of patients admitted but also their risk profiles and infection control needs.

Critically ill cardiac patients in CCUs are prone to complications that are either intrinsic to their cardiac condition (e.g., cardiogenic shock) or are related to advanced therapies (e.g., antithrombotic regimens and mechanical circulatory support). These patients are also frequently treated with complex pharmacological agents such as angiotensin receptor–neprilysin inhibitors (ARNIs) and advanced device therapies like CRT. Although these treatments improve clinical outcomes, they also necessitate prolonged hospitalization, repeated device handling, and intensive monitoring—all of which increase the risk of HAIs. Recent findings by Pătru et al. show that ARNI therapy in CRT-treated patients, especially in non-responders, significantly improves left ventricular function and clinical status [97]. However, this therapeutic complexity underscores the importance of tailored infection control protocols in CCUs, as these patients are more vulnerable to device-associated infections such as CLABSIs, CAUTIs, and VAP.

The findings from this review emphasize that the limited availability of standardized, CCU-specific HAI data hampers efforts to develop tailored infection control protocols. Recognizing the types and frequencies of those infections most commonly observed in CCUs—such as CLABSIs, CAUTIs, and VAPs—should prompt the implementation of stricter device-use policies, early catheter removal protocols, and targeted surveillance programs. Moreover, an awareness of institutional and regional variations in reporting underscores the need for universal adherence to standardized case definitions and documentation systems. Integrating routine HAI audits, checklists for device handling, and cross-disciplinary education into CCU workflows can meaningfully reduce the infection burden, shorten hospital stays, and improve survival outcomes in this high-risk population.

Limitations and Risk of Bias Considerations: A critical limitation of this review is the variability found in study design, data quality, and reporting standards among the included sources. Many studies lacked consistent methodologies, and few performed adjustments for cofounders or included formal risk of bias assessments. Moreover, the reliance on retrospective data and administrative coding in several regions—especially in developing countries—raises concerns about the potential underreporting or misclassification of HAIs. Definitions of infection types and diagnostic criteria also varied significantly, which may affect prevalence estimates and cross-study comparability. These inconsistencies emphasize the need for future studies to adopt standardized surveillance protocols and validated case definitions to enable more accurate benchmarking across countries and care settings.

## 6. Conclusions

Overall, the current literature on HAIs in CCUs remains fragmented and underdeveloped, despite the clear vulnerability of this patient population. While the global data show a wide range of HAI prevalence rates, few studies specifically focus on patients with acute heart failure in CCUs. To address this gap, future research should prioritize large-scale, prospective multicenter studies using standardized definitions and surveillance protocols to better characterize the burden of HAIs in CCUs.

From a policy and clinical perspective, hospitals should consider implementing CCU-specific infection prevention protocols, including device minimization strategies, early catheter removal pathways, structured antimicrobial stewardship programs, and routine HAI audits. Establishing national registries and real-time reporting systems may also improve data accuracy and guide quality improvement efforts. Finally, incorporating infection control training into continuing education programs for CCU staff can foster a culture of safety and help reduce preventable infections, ultimately improving outcomes for high-risk cardiac patients.

As an awareness of the unique challenges in CCU infection control continues to grow, coordinated action across clinical, administrative, and research domains will be essential to protect this critically ill population and elevate the standard of cardiovascular intensive care globally.

## Figures and Tables

**Table 1:** Prevalence of HAIs in patients with cardiovascular diseases from different continents.

Continent	Country	Population	No. of Tested Persons	Prevalence of HAIs	Reference
AMERICA	USA	Cardiac surgery patients	4320	2.8%	Greco G, 2015 [33]
	USA	Cardiac surgery patients	8853	4.2%	Etchill EW, 2022 [34]; Lazar HL, 2022 [35]
	USA	Cardiovascular patients	17,889,852	0.88%	Miller PE, 2019 [29]
	USA	CABG patients	324,085	2.83% pneumonia	Brescia AA, 2017 [39]
	USA	CABG patients	87,318	5.73%	Vogel TR, 2010 [38]
	USA	Cardiac surgery patients	605	21.7%	Kollef MH, 1997 [36]
	USA	STEMI patients	105,184	19.1%	Chehab O, 2020 [40]
	USA	CSICU patients	40,207	1.69% BSI	Gordon SM, 1998 [32]
	Brazil	AMI patients	1227	5%	Grandini LC Jr, 2006 [41]
	Brazil	Cardiac surgery patients	195	22.6%	Ferreira GB, 2020 [37]
	Brazil	IE infective endocarditis patients	151	35.09%	Francischetto O, 2014 [42]
ASIA	6 countries	ECMO patients	2955	31.57%	Lv X, 2024 [51]
	Korea	ECMO patients	150	23.3%	Ko RE, 2020 [51]
	China	Cardiac surgery patients	1360	6.54%	Liu Z, 2021 [44]
	China	AF patients	1059	25.87% HAP	Zhu J, 2015 [49]
	China	Cardiac surgery patients	720	20.97%	Yao X, 2023 [47]
	China	Medical ICU patients	2757	12.1%	Chen YY, 2009 [43]
	China	Cardiac surgery patients	1606	7.8%	Jiang WL, 2018 [48]
	India	Cardiac surgery patients	6864	4%	Sahu MK, 2016 [45]
	Iran	Cardiac surgery patients	610	17–23%	Damavandi DS, 2019 [46]
	Japan	Endocarditis patients	158	33.5%	Kiriyama H, 2020 [50]
AUSTRALIA	Australia	Endocarditis patients	2760	6.4%	Athan E, 2012 [53]
	Australia	Cardiac surgery patients	43,691	0.7–12.4%	Sanagou M, 2016 [54]
EUROPE	Turkey	ECMO patients	126	36.5%	Selçuk ÜN, 2021 [62]
	Switzerland	AHF patients	178	17%	Rudiger A, 2010 [60]
	Norway	CABG patients	2440	5.1%	Berg TC, 2011 [63]
	Spain	Cardiac surgery patients	970	9.2%	Rebollo MH, 1996 [66]
	Spain	ICU acute heart disease patients	69,876	5.1%	Renes Carreño E, 2022 [59]
	Spain	MHS patients	800	15.5%	Pérez-Granda MJ, 2024 [67]
	Spain	Cardiac surgery patients	1097	10.1%	de la Varga-Martínez O, 2021 [68]
	Greece	CVICU patients	172	13.95%	Lola I, 2011 [69]
	Greece	Cardiac surgery patients	2122	5%	Michalopoulos A, 2006 [70]
	Greece	CABG patients	782	2.7	Falagas ME, 2006 [64]
	Greece	CABG patients	360	5%	Rosmarakis ES, 2007 [65]
	Austria	Cardiac surgery patients	195	38.5%	Selçuk ÜN, 2021 [62]
	Netherlands	Cardiac surgery patients	954	19.8–26.2%	Segers P, 2006 [71]
	Italy	Cardiac surgery patients	925	9%	De Santo LS, 2008 [72]
	France	Cardiac surgery patients	7557	1.7%	Le Guillou V, 2011 [73]
	Finland	AHF patients	620	24%	Rudiger A, 2010 [60]
	France	AHF patients	581	20%	Rudiger A, 2010 [60]
	Europe	AHF patients	3580	18%	Nieminen MS, 2006 [61]
	25 European countries	MHS patients	971	4.4%	Hortal J, 2009 [74]
	13 European countries	MHS patients	164	26.8%	Bouza E, 2006 [75]
	17 European countries	MHS patients	11,915	9.9%	Bouza E, 2006 [76]

HAIs = healthcare-associated infections; ICU = intensive care unit; CABG = coronary artery bypass graft; CVC = cardiovascular; STEMI = ST elevation myocardial infarction; AMI = acute myocardial infarction; AF = atrial fibrillation; ECMO = extracorporeal membrane oxygenation; AHF = acute heart failure; MHS = major heart surgery; BSI = bloodstream infection; HAP = hospital-acquired pneumonia.

**Table 2:** Overall prevalence of HAIs in Romania.

Population	No. of Tested Persons	Prevalence	Reference
ICU patients from a university hospital in Western Romania	1596	38.6% in ICU	Axente C, 2017 [79]
Patients from 7 public hospitals in Mures	228,782	0.44% (48.25% in ICU)	Voidazan S, 2020 [17]

ICU = intensive care unit.

## Data Availability

Not applicable.
